# The practice of genomic medicine: A delineation of the process and its governing principles

**DOI:** 10.3389/fmed.2022.1071348

**Published:** 2023-01-12

**Authors:** Julia Handra, Adrienne Elbert, Nour Gazzaz, Ashley Moller-Hansen, Stephanie Hyunh, Hyun Kyung Lee, Pierre Boerkoel, Emily Alderman, Erin Anderson, Lorne Clarke, Sara Hamilton, Ronnalea Hamman, Shevaun Hughes, Simon Ip, Sylvie Langlois, Mary Lee, Laura Li, Frannie Mackenzie, Millan S. Patel, Leah M. Prentice, Karan Sangha, Laura Sato, Kimberly Seath, Margaret Seppelt, Anne Swenerton, Lynn Warnock, Jessica L. Zambonin, Cornelius F. Boerkoel, Hui-Lin Chin, Linlea Armstrong

**Affiliations:** ^1^Department of Medical Genetics, University of British Columbia, Vancouver, BC, Canada; ^2^Provincial Medical Genetics Program, British Columbia Women’s Hospital and Health Centre, Vancouver, BC, Canada; ^3^Department of Pediatrics, University of British Columbia, Vancouver, BC, Canada; ^4^Department of Pediatrics, Faculty of Medicine, King Abdulaziz University, Jeddah, Saudi Arabia; ^5^Clinical Research Informatics, Provincial Health Services Authority, Vancouver, BC, Canada; ^6^Process & Systems Improvement, Provincial Health Services Authority, Vancouver, BC, Canada; ^7^Breakthrough Genomics, Irvine, CA, United States; ^8^Women’s Health Research Institute, British Columbia Women’s Hospital and Health Centre, Vancouver, BC, Canada; ^9^Khoo Teck Puat-National University Children’s Medical Institute, National University Hospital, Singapore, Singapore

**Keywords:** genomic medicine, precision medicine, distributed cognition, workflow optimization, integrated care

## Abstract

Genomic medicine, an emerging medical discipline, applies the principles of evolution, developmental biology, functional genomics, and structural genomics within clinical care. Enabling widespread adoption and integration of genomic medicine into clinical practice is key to achieving precision medicine. We delineate a biological framework defining diagnostic utility of genomic testing and map the process of genomic medicine to inform integration into clinical practice. This process leverages collaboration and collective cognition of patients, principal care providers, clinical genomic specialists, laboratory geneticists, and payers. We detail considerations for referral, triage, patient intake, phenotyping, testing eligibility, variant analysis and interpretation, counseling, and management within the utilitarian limitations of health care systems. To reduce barriers for clinician engagement in genomic medicine, we provide several decision-making frameworks and tools and describe the implementation of the proposed workflow in a prototyped electronic platform that facilitates genomic care. Finally, we discuss a vision for the future of genomic medicine and comment on areas for continued efforts.

## 1. Introduction

*DNA provides a language that prefigures possibilities and constraints for both the development and the present state of homeostasis of cells, organs, and individuals, while also encoding their phylogenetic history.*

—Barton Childs

—Genetic medicine: A logic of disease

**FIGURE 1:**
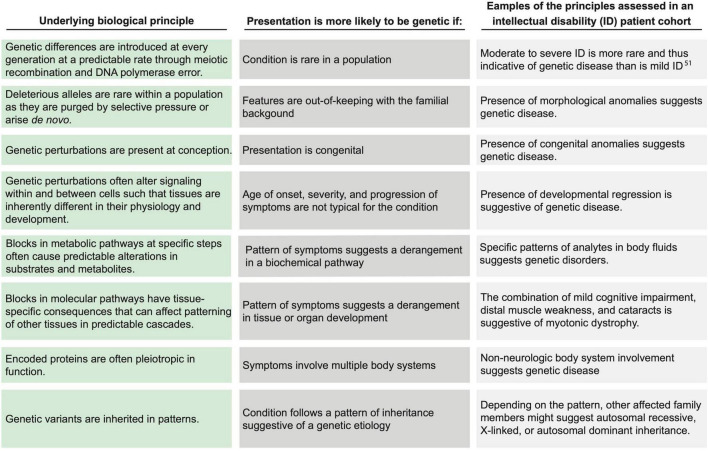
Applying biological principles to assess the likelihood of genetic disease. Within the biologic principles of disease **(left column)**, certain clinical attributes **(center column)** increase the likelihood of a genetic disease. The **(right column)** lists examples of clinical representations of such biological principles for individuals with intellectual disability. Each example may be representative of more than one biological principal. Drawn from current understanding of evolutionary biology, these principles were synthesized and defined by the Provincial Medical Genetics Program (PMGP) clinical genomic specialists (CGSs), and the examples were drawn from their clinical practices.

The principle of the gene ties each species and individual to phylogenetic history, ontogeny, and environmental homeostasis. The introduction of genetic variation disrupts homeostasis with the environment. The resultant environmental incompatibility is fundamental to the concept of disease and applies to rare monogenic disorders as well as to common, multifactorial diseases. Because disease obeys a logic imposed by the attributes and constraints of genetic variability (Figure 1), medicine is, consequently, informed by the evolution of the human species as well as by each patient’s unique genetic constitution, which is a test of genetic attributes within the selective pressures exerted by nature. Genomic medicine codifies these integrative principles to guide the diagnosis and management of patients.

Publicity, rapid technological advances, accumulation of genomic knowledge, declining costs, and the availability of specific and effective biological and gene-based therapies are creating increasing demand for genome-informed personalized medicine (1–5). Targeted genetic testing, whole exome sequencing (WES), and whole genome sequencing (WGS) are increasingly recognized as enablers of precision medicine and are readily utilized to discover disease-contributing variants (2). These technologies enable comprehensive molecular profiling that overcomes the limitations of medical genetics, in which a phenotypic diagnosis was often provided in the absence of molecular findings. Despite this, however, the practice of genomic medicine remains limited by a lack of infrastructure, inadequate cost amortization, and a paucity of genomically educated personnel. These limitations contribute to the “diagnostic odyssey” and more recently to the “therapeutic odyssey” (6). The latter is a particularly important consideration for genetic disorders as the timing of initiation of therapy can profoundly influence the ultimate outcome of patients.

Current clinical practice models are not designed to capture, query, or manage genomic data, to handle the uncertainty inherent in variant interpretation (quantify phenotypic evolution over a lifetime), or to integrate and compute the physiologic, epigenomic, and environmental context of genomic variants for a given patient (2). Consequently, the data from genomic testing are rarely integrated within healthcare and seldom re-patriated from private sector genetic testing laboratories to build local databases or to contribute to the collective cataloging of genomic variation. Such databases are essential to interpret genomic variation, to build the infrastructure for future precision medicine initiatives, and to enable utilization of genomic data over the patient’s life.

Herein we generate an overarching vision and process for genomic medicine practice by framing it within the principles of evolutionary medicine and cultural principles of utility. This framework, which was tested within the Provincial Medical Genetics Program (PMGP) of British Columbia, is neither a summary of Canadian experience nor official guidelines for care. It is a set of globally applicable philosophical principles defining the requisite tasks, outcomes, decisions, and key players. The work highlights the need for formal training of clinicians in genomic medicine, i.e., clinical genomic specialists (CGS).

## 2. Methods

### 2.1. Generation of the genomic medicine process map

**FIGURE 2:**
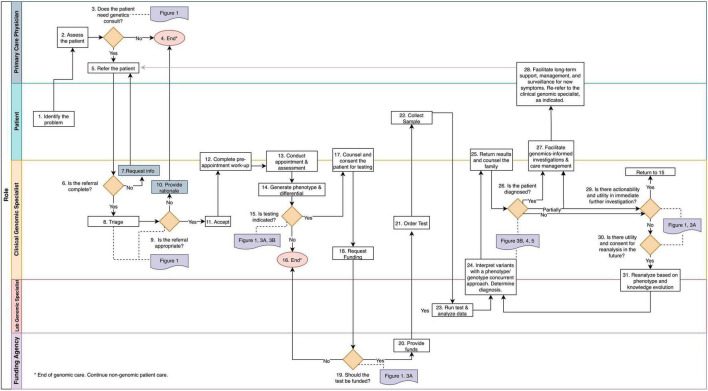
The genomic medicine process map. This workflow illustrates genomic medicine practice, which differs from medical genetics in that molecular profiling is required for diagnosis. The process begins when a patient or the PCP identifies a health concern. Rectangles denote tasks and orange diamonds denote decision points. Swim lanes correspond to the player responsible for completion of the tasks. References to the frameworks and guidelines presented throughout the manuscript are in purple and are connect to the corresponding decision point with a dotted line. The tasks and decision points are numbered to reference text descriptions (Supplementary material section 1); this numbering does not always reflect a linear sequence of steps due to decisional loops. Endpoints are denoted by pink circles.

To identify and document clinical tasks and decision-points from referral to discharge, we mapped operating procedures within three PGMP genomic medicine practices and discussed procedures and workflows with medical geneticists, genetic counselors, program managers, and clerical staff (Figure 2 and Supplementary material section 1). In the context of prior thought, (7–11) these clinicians synthesized the biological principles that govern their decision-making in daily practice (Figure 1). The resulting workflow was used to design, prototype, and implement an electronic data management system as a quality improvement initiative within the PMGP of British Columbia.

### 2.2. Implementation of the genomic medicine practice process into REDCap

Having mapped the processes of genomic medicine practice and defined the logic of the biological and utilitarian practice decisions, we built the genomic medicine practice process into an instance of the research electronic data capture (REDCap) system and implemented this infrastructure as a quality improvement initiative within the PMGP. Previously identified data inefficiencies and vulnerabilities were addressed within the REDCap platform by building instruments to facilitate referral management, triage, patient assessment and phenotyping, testing coordination, and results tracking (Supplementary material section 2). During the first year of use, the implemented platform was iteratively optimized with input from over 50 clinicians and staff and was used to manage 10,000 patient records.

## 3. Results

The decisions and tasks that enable the practice of genomic medicine are depicted in the Genomic Medicine Process Map (Figure 2). Process mapping of genomic medicine practice for purposes of diagnosis identified four stages of care: (1) phenotypic assessment and generation of a prior risk for a genetic disease, (2) evaluation of the clinical utility of genomic testing, (3) analysis and interpretation of genomic variation, and (4) genome-informed patient management.

### 3.1. Genomic medicine practice is collaborative

The process of genomic medicine practice engages five key players: the patient, the principal care provider (PCP), the clinical genomic specialist (CGS), the laboratory genomic specialist (LGS), and the payer. The roles and responsibilities of each entity are likely assumed by a team (Table 1). The CGS delivering genome-informed care might be a team of a specialist physician, genetic counselor, and support staff, and, in the context of this work, the LCS predominantly represents those engaged in the interpretation and reporting of genomic variation while recognizing that such is built on the analytical validity of the data generated by the laboratory.

**TABLE 1:** Five key players in genomic medicine.

Role	Description	Responsibilities
Patient	The patient and their family members or guardians are the primary drivers and informants of care. Their needs and priorities are placed at the center of care.	● Communicate care needs, medical history, concerns, priorities, and values to care providers. ● Engage as an active participant in decision-making, investigation (current and future), and care management. ● Inform care team of changes in health during or after testing. ● Request appropriate reassessments for diagnosis and management.
Principal care provider (PCP) (31, 32, 34, 46–48, 55)	The PCP is the clinician who provides longitudinal (ongoing) care and coordinates evaluations and services. The PCP plays a critical role in the continuation of genomics-informed care after testing.	● Respond to initial patient concerns. ● Assess the risk of genetic disease and refer the patient for genomic services. ● Contextualize genomic investigations and findings for long-term care and management.
Clinical genomic specialist (CGS)	The CGS is a clinician with a comprehensive understanding of the biological mechanisms of genetic disease, Bayesian decision-making, and genomics. The involvement is typically that of a consultant. The CGS integrates genomic knowledge, patient needs and phenotype, and variant information to interpret genome-wide sequencing (GWS) results. Some tasks in the interpretation of genomic data may overlap with the role of the LGS.	● Apply biological rationale in assessing the likelihood of genetic disease and the suitability of genomic testing, advocate eligibility to funders, and codify phenotype for testing. ● Educate, counsel, and consent the patient for genomic testing. ● Collaborate with the laboratory, the PCP, and the patient in clinically contextualizing genomic findings; this might include assessing the need for further investigations. ● Communicate interpretation of variants to patients and PCPs with a clear directive on genomics-informed follow-up care.
Laboratory genomic specialist (LGS)	The “LGS” encompasses the sequencing laboratory that produces genomic data and those that interpret, classify, and report the pathogenicity of genomic variants. The LGS collaborates with the CGS in the clinical contextualization of GWS findings.	● Ensure the quality and validity of genomic testing and analysis technologies. ● Integrate information sources to assess and prioritize variants by using standardized guidelines. ● Communicate test findings to the CGS with evidential transparency and engage in collaborative interpretation.
Funder	The funder assesses the utility of GWS in the context of financial constraints and allocates funding accordingly.	● Derive guidelines from biological and utilitarian frameworks for allocation of genomic testing. ● Assess funding eligibility on a case-by-case basis. ● Assess cost-effectiveness of testing programs.

### 3.2. Genomic medicine practice is governed by biological and statistical principles

**FIGURE 3:**
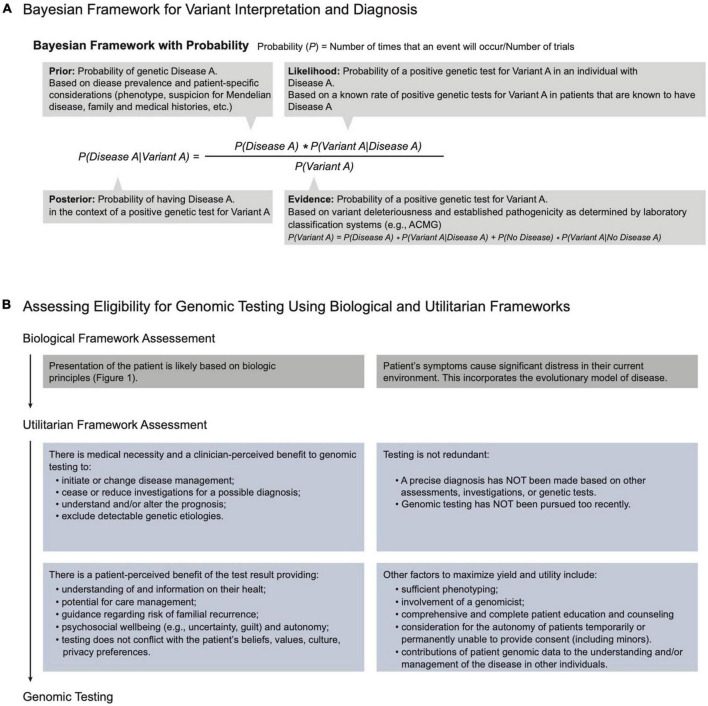
Principles and frameworks governing genomic medicine. **(A)** Bayesian framework: A thought process for variant interpretation and diagnosis. Bayes theorem quantifies the probability of “A” being true given some evidence “B.” Applying this framework to genomic diagnosis, we can assess the posterior probability of a patient’s disease (Disease A) being caused by a genetic variant (Variant A) by dividing the product of the prior probability of Disease A and the likelihood of the variant in the context of the disease. As this formula serves to guide a thought process, numerical input and quantification of the probabilities are not required. **(B)** Assessing eligibility for genomic testing using biologic and utilitarian frameworks. Given that assessment of biological principles suggests that a patient has an increased likelihood of genetic disease (Figure 1), application of a utilitarian framework allocates resources to enable testing that accomplishes societal expectations of benefit, impact, and utility for the patient, the family members, and the payer.

Within the process map, the decisions and tasks for referral, triage, assessment, testing, and management are grounded in a biological framework that assesses the likelihood of a unifying genetic cause or a contributing genetic factor. The biological principles and the corresponding clinical attributes of the patient are depicted in Figure 1. The clinical attributes of the patient (i.e., the phenotype), which are ascertained through the process of medical and family history taking, examination, and investigation, are evidence in a Bayesian likelihood framework (Figure 3A and Supplementary material section 4A). Currently, because of the lack of population and aggregate data, this framework is a semi-quantitative approach providing logical guidance, rather than a fully quantitative approach.

In the Bayesian approach, the clinical attributes provide a likelihood of genetic disease, and the specificity of the features suggest the likelihood of a particular disease. Together these represent the *prior* probability. Clinically, these likelihoods reflect the physician’s acumen in differentiating individuals more likely to benefit from genomic testing and the physician’s suspicion for a specific genetic diagnosis prior to genetic testing.

The *prior* informs the probability of a variant’s pathogenicity and thus the yield of genetic testing in the patient (Figure 3A; 12, 13). The biological framework governing genomic medicine is thus intimately linked with Bayesian logic and relies on the CGS to apply the biological framework with Bayesian logic when assessing the likelihood of genetic disease, selecting testing, determining diagnosis, interpreting results, and managing patients.

### 3.3. Genomic medicine is constrained by societal resources

Given that resources in every society are limited, a utilitarian framework allocates resources such as personnel, cognitive investment, and testing for genomic medicine practice (Figure 3B). Utilitarian principles assess the expected benefits to the patient, family members, payer, and society in order to maximize service and minimize opportunity costs (14). Applied concurrently with biologic principles, this approach optimally prioritizes those most likely to benefit from genetic testing, i.e., achieve a diagnosis or precise therapy (Figures 1, 3B; 15, 16). Genomic medicine is complimentary to societal utilitarianism in that rapid and precise diagnoses lead to more informed care and better utilization of resources.

### 3.4. Stage 1: Initiation of genomic care, phenotypic assessment, and generation of prior risk of a genetic disease

This stage pertains to steps 1–14 of Figure 2. A phenotype-first approach, which identifies those most likely to have genetic disease and benefit from testing, remains common in genomic medicine practice. Evidence gathered through collaboration of the patient, PCP, and CGS informs the likelihood of a genetic disease and identifies potential diagnoses prior to genomic testing.

As for many clinical processes, care begins either when a patient or a family member of an affected individual communicates a concern to the PCP or the PCP notes a concern on a routine health check. Although dependent on the medical care organization within a society, in many societies the PCP determines whether the clinical presentation meets a threshold of perceived probability and importance that warrants a genomic assessment. Whether patient or PCP initiated, referrals to a CGS are typically framed as requests for further expertise or for a service to address a clinical question. In the latter scenario, the PCP refers the patient to a CGS when (i) the patient’s clinical presentation warrants evaluation that is outside of the PCP’s knowledge of genomics, (ii) genetic investigation is warranted and test selection and/or result interpretation is outside of PCP expertise, or (iii) genetic counseling is warranted and is outside of PCP expertise. CGS referrals requesting diagnostic services must contain (i) sufficient documentation of the clinical attributes suspicious for genetic disease as per biologic principles and (ii) a referral question that is potentially resolvable by CGS assessment and/or by genetic testing.

Electronic collection of additional information, consent forms, pre-appointment intake questionnaire, and possibly photographs from patients prior to the clinical appointment empowers informed, efficient, and effective patient-provider interactions and increases patient self-advocacy and satisfaction (17, 18). Prior collection of medical, developmental, and family histories and the review of systems increase the efficiency of CGS clinic visits and might suggest a phenotypic pattern indicative of specific diagnoses that warrants a targeted evaluation.

### 3.5. Stage 2: Evaluation of the clinical utility of genomic testing

This stage pertains to steps 15–22 of Figure 2. Following the assessment and an evaluation of the likelihood of a genetic disease, the CGS considers the potential clinical utility of genomic testing. For transparent and objective determination of utility, we generated and piloted a tool for patients with intellectual disability (Supplementary material section 3). This tool demonstrates the thought process to assess whether testing is indicated for a patient (step 15 in Figure 2) and how missing clinical history or patient features skew perceptions of the potential utility of testing. Assessment by the CGS often occurs at a single time or over a short interval. Awareness that perceived utility of testing fluctuates with phenotypic evolution is, therefore, critical to understanding negative test results for those whose symptoms resolve and for those who need re-referral when symptoms persist or progress (Supplementary material sections 4A, B).

Test selection is informed by disorders that the CGS considers as possible causes for the clinical presentation, by the molecular mechanisms by which these disorders arise, and by the laboratory test characteristics. A non-specific phenotype has a broad differential and often warrants genomic profiling (WES or WGS). For example, appropriate testing for a patient with infantile hypotonia includes WES and assessment for *SMN1* deletion, expansion of the CTG trinucleotide repeat in the non-coding region of *DMPK*, and *SNRPN* methylation; this highlights the need to understand the molecular mechanisms of disease and the technological limitations of each test. As a result of its comprehensiveness, exome (and genome) sequencing is a cost-effective first tier test for many indications (15, 16, 19), although an exome-first approach does conflict with some jurisdiction-imposed tiered genetic testing strategies (e.g., requiring a chromosomal microarray prior to funding whole exome sequencing).

A patient’s decision to pursue testing is informed by perception of the test’s utility (Figure 3B). Patients understand and assess utility as presented by the CGS *via* the process of informed consent. This includes an appraisal of the possible benefits, risks, limitations, results, and implications of the genetic findings to the patient and family members (5, 20). Additionally, disclosure of results is guided by understanding a patient’s values regarding the utility of testing.

The decision whether to fund a proposed test is made by the payer, i.e., the patient or an insurer. The payer assesses precedent and considers a utilitarian framework to decide whether to allocate limited resources. Providing the evidence of medical necessity is the responsibility of the CGS. Pre-determined genomic testing eligibility criteria or case-by-case utility assessments guide payer decisions (21). Decisional transparency by payers is essential to ensure equity and advocacy, which is needed when testing is supported by the biologic principles but the payer declines funding (14, 22).

### 3.6. Stage 3: Analysis and interpretation of genomic variation

**FIGURE 4:**
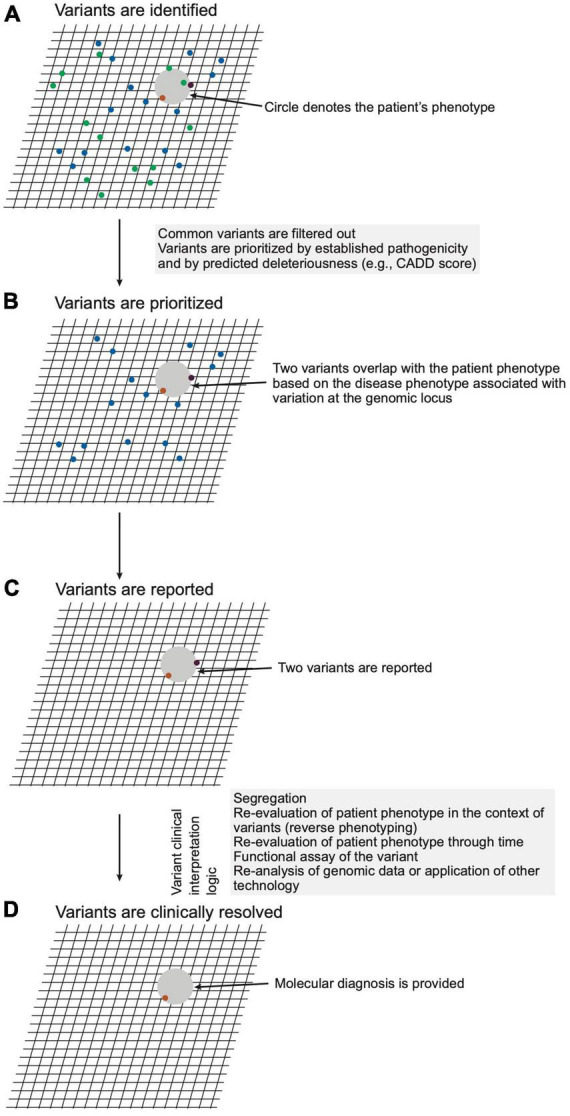
Schematic representation of variant analysis and resolution. **(A)** A hypothetical patient phenotype (gray circle) is plotted on an imaginary grid in relation to disease phenotypes associated with genomic loci within which the patient carries variants (ochre, purple, green dots). **(B)** Given that the patient has a rare disorder, variants with high unaffected population frequencies (green dots) are eliminated from consideration. **(C)** The remaining variants are filtered considering precedent (prior association with phenotype), variant type (e.g., non-synonymous, frameshift, premature stop), and *in silico* prediction of deleteriousness. The remaining two variants, which meet a threshold of potential deleteriousness and occur in loci associated with diseases phenotypically overlapping that of the patient, are prioritized and reported by the laboratory. **(D)** The CGS constructs a posterior probability for a diagnosis through review of the reported variants for evidence that variation of the genomic loci cause disease, for concordance of the patient phenotype with that of the diseases, and for genetic, molecular, and biochemical congruence of the variant with the disease mechanism. For this hypothetical patient, the clinical genomic specialists (CGS) judges one variant (ochre dot) a sufficient sole molecular diagnosis.

This stage pertains to steps 23–24 of Figure 2. The next stage of genomic medicine practice encompasses genomic analysis and interpretation of the variation to evaluate potential molecular diagnoses. The LGS ensures the quality of sequencing, alignment, genotyping, annotation, variant filtering, classification, and reporting. Filtering and prioritization of variants for interpretation are guided by the patient phenotype and by the variant attributes (Figure 4 and Supplementary material section 5). Prioritizing variants based on the concordance of the patient phenotype with that of the disease(s) arising from variation at a locus optimizes the sensitivity and specificity of genetic testing (23, 24). Phenotype is often provided as a CGS-curated list of pertinent positive and negative signs, traits, and symptoms using the vocabulary of Human Phenotype Ontology (HPO). As ontological entities with information content and relationship, HPO terms provide a standardized language for communication, enable software-prioritization (e.g., Exomiser and LIRICAL) of genomic variants and facilitate standardized electronic exchange of phenotype as phenopackets (25, 26). In lieu of selecting HPO terms, natural language processing is increasingly used for comprehensive phenotype recognition from medical notes to ensure a high recovery of clinical keywords for downstream genetic analysis (e.g., Virtual Geneticist). These terms are commonly supplemented with prose describing the natural history and family history.

Variant attributes considered include frequency in affected and unaffected populations, potential deleteriousness of the variant, congruence with the established disease mechanism, conservation of the reference nucleotide and amino acid across species, and segregation of the variant with the disease in question (17). Variants are classified by the LGS according to the criteria of professional bodies such as the American College of Medical Genetics (ACMG) (17). The consistent application of these criteria should result in a variant receiving the same laboratory classification regardless of the LGS completing the analysis or the clinical features of the tested individual.

Regarding which variants to report, the LGS is guided by expert opinion and professional consensus, i.e., published guidelines (22). For example, the LGS generally does not report a single pathogenic allele in a gene that causes recessive disease, unless there is sufficient evidence to suggest concordance between the patient’s phenotype and the disease in question (Figure 4 and Supplementary material section 5). Similarly, the LGS generally limits reporting of variants of uncertain significance to those with the potential to cause diseases concordant with the patient phenotype. In both situations, the extent of phenotypic concordance needed for reporting of a variant is left to the judgment of the LGS. Within the ideal state of collaboration, the CGS provides a sufficiently detailed phenotype to allow optimal variant prioritization, and the LGS, reciprocally, provides evidential transparency for reported variation.

**FIGURE 5:**
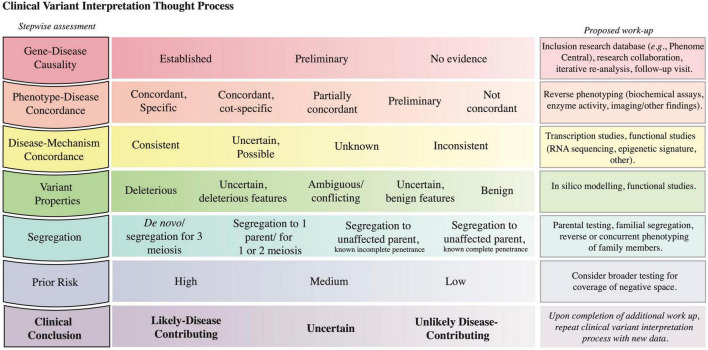
Clinical variant interpretation logic. The left column includes the key lines of evidence to consider in variant interpretation; it starts with assessment of gene-disease causality and progresses to the integration of prior risk. A detailed description of the principles establishing gene-disease association, phenotype-disease concordance, disease-mechanism concordance, and variant deleteriousness, and a Clinical Variant Analysis Tool were reported previously to facilitate the capture and synthesis of these multiple lines of logic in assessing causality of variants (27). A depiction of how phenotype specificity informs the posterior probability that Variant A contributes to the patient presentation is presented in Supplementary material section 6. The prior risk refers to the likelihood of the Disease A in the patient, as determined before the initiation of genomic testing (Figure 3A). The middle column lists possible assignments for each row; the left-most option is most supportive of variant contribution to disease, whereas the right-most option is least supportive of variant contribution to disease. The third column highlights methods that can be pursued to collect further evidence to inform the corresponding assessment. The last row denotes the clinical conclusion based on the integration of the other rows (56).

Given the nuances of genomic testing and variant reporting, the CGS contextualizes the laboratory-reported variants to establish a diagnosis. To formulate a clinical conclusion, variant interpretation considers factors inherent to a variant in parallel with the existing knowledge about the locus (gene-disease causality, disease-mechanism concordance) and the patient’s clinical presentation (phenotype-disease concordance, segregation) (Figure 5 and Supplementary material section 6 for case examples). The evidence from this logical framework modifies the *prior* of the Bayesian equation (Figure 3A) such that the posterior probability informs the likelihood that a variant, *Variant A* in *Locus A*, contributes to the patient’s presentation.

Completion of the logical framework often requires further evaluation or testing such as segregation, reverse phenotyping, or functional testing. For instance, variant segregation in the family determines if the identified variants (i) for a recessive disorder are in *cis* or *trans*, (ii) arose *de novo*, or (iii) segregate with the phenotype in the family. Segregation data requires an understanding of the penetrance and expressivity of a condition; for example, when the locus in question has previously shown incomplete penetrance for a disease, segregation to unaffected individuals in a small pedigree is insufficient to prove that a variant is benign. The likelihood that the variant in question contributes to a particular disease is modified by assessment of the patient for phenotypic concordance with the disease in question and by functional assays (Figure 5 and Supplementary material section 6). Of note, even if these studies do not lead to variant reclassification within the ACMG guidelines, the data might be sufficient for the CGS to decide whether a variant contributes to the patient’s presentation and if contributing, whether it contributes a full or partial explanation.

Overall, approximately 25–30% of exome sequencing results require further testing by a variety of methods to support the diagnostic process (18, 27). Of variants with ambiguous pathogenicity after these additional evaluations, most will not require further patient intervention until their significance is clarified; current understanding predicts that over time most of these variants will be re-classified as benign (20). Rarely, the CGS might judge ambiguous variants actionable and make management recommendations. Appropriate patient counseling and collegial consensus in these rare scenarios is paramount.

A challenge for the diagnosis of rare disorders is determining whether features previously unobserved with a disease represent an expansion of the disease phenotype or a second disease. Multiple molecular diagnoses, which are predicted to occur in 14–26% of probands (21, 28), are under-reported due to parsimony-based analytical pipelines and cognitive bias (27). Cataloging of the phenotype associated with each variant by submission to repositories like ClinVar and by publication of case and cohort reports facilitates the phenotypic expansion of each genetic disease. The documentation of the clinical variant interpretation within databases like ClinVar complements the ACMG classifications and builds distributed cognition.

### 3.7. Stage 4: Genome-informed patient management

This stage pertains to steps 25–31 of Figure 2. Regardless of the outcome of genomic testing, collaboration between the referring provider, the CGS, and the patient ensures the best care plan and follow-through on management recommendations. Patients and families diagnosed by genetic testing commonly report uncertainty about the meaning of a genetic diagnosis (29) despite being informed by the CGS of the diagnosis and its molecular etiology, clinical features, and implications (e.g., recurrence risk). To assist the patient’s adjustment to the diagnosis, the CGS often coordinates the implementation of medical care by other care providers, facilitates genetic counseling, and potentially pursues cascade testing in family members. When specialty clinics are unavailable, the PCP often coordinates the long-term support and management (30–35).

When interpreting negative results, the CGS considers the patient’s *prior* probability of genetic disease (stage 1) and the context of testing (including the perceived utility by the patient, stage 2) to determine whether the result represents a true or a false negative (Supplementary material section 4B). For instance, a negative genetic test result might increase the likelihood of an alternate non-genetic diagnosis and is, therefore, informative for diagnosis, management, and recurrence risk. Given that the current diagnostic yield of WES is only 35–55% among patients with traits suggestive of a genetic etiology (36–38), the CGS might remain suspicious of an underlying genetic etiology and suspect a false negative result. False negatives can occur due to limitations in knowledge or in technology.

For individuals suspected to have a genetic disorder but who do not have a molecular diagnosis, genetic counseling assists the patient/family’s adjustment to the absence of an objective explanation (6). Additionally, their awareness that the additive and iterative capture of phenotype evolution (including resolved features) promotes constructive engagement in the diagnostic odyssey. Expansion of medical knowledge also promotes diagnostic resolution such that after 1 year, new genomic locus-disease or phenotype-disease associations increase exome diagnostic yield for Mendelian disorders by approximately 10% (39–42). Concurrently or alternatively, the patient characteristics might be sufficiently compelling that the CGS offers additional clinical testing or research-based investigations. Examples of such clinical testing include whole genome sequencing or testing of another tissue to identify a mosaic variant. Examples of research-based investigations include long-read sequencing, *de novo* genome assembly, methylome analysis or transcriptome analysis (18). Also, exchange of suspicious variants (PhenomeCentral^1^, GeneMatcher^2^, or MatchMaker exchange^3^) facilitates novel disease gene discovery through generation of cohorts of patients with similar phenotypes and variants.

### 3.8. Limitations and challenges in genomic medicine

Mapping the genomic medicine process across the PMGP of British Columbia identified processes that limited genomics-informed healthcare in each of the four stages of genomic care (Table 2). These limitations to genomic medicine practice are present globally to varying degrees and with cultural nuances. An example of a culturally nuanced limitation is the differences among payers (43).

**TABLE 2:** Challenges and limitations in genomic medicine.

Subsection	Players	Challenges
Phenotypic assessment and generation of a prior risk for a genetic disease	Patient, PCP, CGS	Objective capture and quantification of the pertinent clinical attributes
Evaluation of the clinical utility of genomic testing	Patient, CGS, payer	Quantification of eligibility; standardized presentation of biologic suspicion; lack of decisional transparency by the payer; lack of outcome data (esp., patient perceived benefit) to inform utility assessment
Analysis and interpretation of genomic variation	CGS, LGS (+patient if further evaluation is needed)	Limitations of HPO terms; individualized evaluation plans; lack of transparent reporting; consistency in the interpretation and classification of variants; incomplete penetrance.
Genome-informed patient management	CGS, PCP, patient	False negatives; lack of evidence-based disease management guidelines

### 3.9. The use of an electronic platform facilitates genomic medicine and addresses challenges

Because genomic medicine relies on an evolving collaboration of patients, PCPs, CGSs, LGSs, and payers, its practice necessitates distributed cognition (i.e., knowledge embedded in objects, individuals, and tools) and integrative technology that enables facile data capture, sharing, management, and analysis. As a quality improvement initiative, the PMGP of British Columbia prototyped an electronic platform for genomic medicine to identify key functions facilitating genomic medicine practice. The customized REDCap electronic data management platform facilitated CGS-patient communication, provided secure data (phenotype, genotype, family history) storage, enabled triage through conditional logic, generated standardized reports, improved data management and querying, and automated data transfer. Replacing multiple *ad hoc* methods of data collection (i.e., paper and Excel spreadsheets), the electronic platform introduced workflow efficiencies. Furthermore, a patient-facing module removed barriers and allowed secure sharing of medical information, photographs, and consent forms. Using customized, automated emails, the platform reduced clerical tasks and achieved a completion rate of 89% for the pre-appointment questionnaire and of 77% for the release-of-information consent form. To standardize funding applications for genomic testing, eligibility and phenotyping instruments captured and communicated pertinent patient details to the payer. This expedited funding approval and access to testing by several weeks. In addition, the capture of information (phenotype, testing eligibility, genomic sequencing results, and outcomes) across a patient’s journey identifies patient cohorts with unique care needs, promotes sharing of cognitive resources in the interpretation of variants, refines testing criteria to maximize utility, and facilitates assessment of quality improvement. The platform is a prototype that will require further development and adaptation to meet the needs of each health system.

## 4. Discussion

We describe a process for genomic medicine that is generalizable and robust to differences in governance, healthcare system, practitioners, and patient populations and applied this to genomic medicine practice within the PGMP. The generalizability and robustness inherent to the process arise from a foundation in the biological or evolutionary origins of *Homo sapiens* and the globally shared principle of societal utilitarianism. The mapping of genomic medicine practice illustrates achievement of precise, proactive, and accessible care through integration of multiple data streams *via* distributed cognition.

Since the completion of the Human Genome Project in 2003, there have been significant strides in understanding human evolution, development, and physiology and in applying this knowledge to medicine. As a result of rapid technological advances and improvements in variant interpretation paradigms, society has established much of the necessary infrastructure to successfully test, diagnose, and counsel patients with rare genetic diseases. The number of medical subspecialties implementing genomic testing in patient care is growing. In response to this, there is a need for educational initiatives that improve general clinical genomic literacy among PCPs and that streamline access of appropriate patients to genetic care pathways (41, 44–48). Building collaborative support networks for result interpretation reduces local knowledge gaps, facilitates the integration of the PCP into the genomic medicine process, reduces strain on genetics services, and minimizes wait-times for patients (14, 19). The distribution of genomic knowledge and expertise closer to primary care enables the medical community to achieve diagnosis and personalized care management sooner in a patient’s health odyssey.

Precision medicine is an emerging healthcare paradigm that, unlike the current one-size-fits-all approach, optimizes care management using an individual’s genetic information. Precision medicine delivery relies on (1) the ability to access an individual’s genetic profile to understand the molecular mechanism of the disease and (2) the therapies that target the identified molecular mechanism and that can be delivered *via* standardized vectors. Genomics-informed diagnoses will have increasing impact on health management given the rise in innovative therapies and preventive interventions with molecular bases. Data produced from the uptake of the genomic medicine workflow (Figure 2) and associated tests serve to define needs in genetic therapy development and to assemble patient cohorts for clinical trials. As the knowledge acquired from rare disease diagnostics in the present accumulates and is applied at the population-level, we foresee a transition to general precision medicine and expansion of integrated molecular profiling to include genomic, epigenomic, transcriptomic, and metabolomic data. The CGS’s role will evolve to include variant interpretation in the context of polygenic and oligogenic disorders and counseling on the likelihood of a health or disease trajectory.

Future work should explore the integration of genomic medicine with precision medicine and precision therapy processes. Healthcare systems will need to adopt secure and accessible storage of clinical and genetic information as structured, discrete attributes to enable iterative re-analysis of genomic data and to provide automated genotype-driven best practice guidelines, decision-support tools, and alerts (49–54). The required coordination and reliance on technology demands an investment from healthcare systems that is rationalized under both biologic and utilitarian principles. Additionally, as there is no precedent for re-contacting and counseling patients regarding the ongoing use of genomic data to inform medical management across the lifespan, the transition to precision medicine is ethically and logistically challenging.

In summary, genomic medicine practice is founded on universal principles that enable its sustainable and scalable practice in all cultures and health systems with the requisite personnel and technical infrastructure (44, 45). The application of biologically and evolutionary-driven genetic investigation maximizes the utility of genomic testing. Obstacles to the implementation of genomics in medicine can largely be overcome through leveraging global distributed cognition.

## Data availability statement

The original contributions presented in this study are included in the article/Supplementary material, further inquiries can be directed to the corresponding author.

## Ethics statement

The requirement for ethics approval for this study was waived by the University of British Columbia and the Children and Women’s Health Centre of British Columbia Research Ethics Board as the study evaluates deidentified data for quality improvement. Written informed consent was obtained from the individual(s), and minor(s)’ legal guardian/next of kin, for the publication of any potentially identifiable images or data included in this article.

## Author contributions

LA, MS, and RH led the clinical review that culminated in the process mapping and REDCap application development. AE, AM-H, AS, CB, EAl, EAn, HL, H-LC, JH, JZ, KSa, KSe, LA, LC, LL, LP, LW, ML, MP, MS, NG, PB, SHy, SHa, SI, and SL contributed to the development of the process map for genomic medicine practice. FM, JH, LA, LS, and SHu developed the REDCap applications. Iterative refinement of the REDCap genomic care application was done through collaboration with AS, KSa, KSe, LS, MS, EAl, EAn, SHa, RH, SHu, SI, FM, MP, AE, CB, H-LC, JH, and NG collaboratively created the concept and content of the manuscript. All authors contributed to the article and approved the submitted version.

## Footnotes

1
https://www.phenomecentral.org

2
https://genematcher.org

3
https://www.matchmakerexchange.org
