# Supplementary material for: The practice of genomic medicine: A delineation of the process and its governing principles
Source: Front Med (Lausanne). 2023 Jan 12;9:1071348. doi: 10.3389/fmed.2022.1071348 (PMC9877428; doi:10.3389/fmed.2022.1071348)
Supplement: Supplementary file 1 [file Data_Sheet_1.docx]

***Supplementary materials***

**The Practice of Genomic Medicine: A Delineation of the Process and its Governing Principles**

**Table of Contents**

[Supplementary Material Section 1. Description of the Genomic Medicine Process Map (Figure 2) 2](#_Toc122249375)

[Supplementary Material Section 2. Prototyping an Electronic Platform for Genomic Medicine Using REDCap 4](#_Toc122249376)

[Supplementary Material Section 3. Genomic Testing Eligibility Assessment Tool 6](#_Toc122249377)

[Supplementary Material Section 4A. Integration and evolution of biologic evidence across the process serves to determine the probability of a genomic diagnosis 9](#_Toc122249378)

[Supplementary Material Section 4B. Testing in the context of phenotype evolution. 11](#_Toc122249379)

[Supplementary Material Section 5. A grid representation of locus/disease-patient phenotypic concordance and specificity. 12](#_Toc122249380)

[Supplementary Material Section 6. Clinical Variant Interpretation Logic Applied 13](#_Toc122249381)

[REFERENCES 16](#_Toc122249382)

# Supplementary Material Section 1. Description of the Genomic Medicine Process Map (Figure 2)

1. The patient or a family member identifies a health issue that is sufficiently concerning or disruptive to seek the medical attention of the principal care provider (PCP) or an equivalent.
2. The PCP collects more information and performs a basic clinical assessment.
3. The PCP assesses the likelihood that the patient has a genomic condition (Figure 1 and 3A) and the necessity of involving a clinical genomic specialist (CGS) based on complexity.
4. Should the PCP conclude that the disease etiology is unlikely genomic, the PCP continues to provide non-genomics-informed care. Should the PCP conclude that the disease etiology is likely genomic and that the investigative needs are relatively simple (e.g., requiring a chromosomal microarray or Fragile X testing), the PCP pursues these tests. If these initial tests are non-diagnostic, the referral to a CGS can be reconsidered.
5. Should the PCP conclude that the disease is likely genomic and that the investigative needs are complex, then the patient is referred to a CGS (Supplemental Material 3. Genomic Testing Eligibility Assessment & Phenotyping Tool). A standardized referral, ideally completed in electronic format, collects information about the patient’s medical and family history. The referral must have a clear clinical question or objective. Indications for testing and Human Phenotype Ontology terms should be included with supporting clinical documentation (e.g., developmental assessments, imaging reports, etc.)
6. The CGS first evaluates the clarity and completeness of the referral.
7. If the information is insufficient, the CGS requests additional information from the PCP.
8. The CGS triages the referral to determine the appropriate involvement of a CGS in the patient’s care.
9. In the triage, the CGS considers the underlying biological principles when assessing the likelihood of genomic disease (Figure 1).
10. If involvement of the CGS is not necessary, the triage team promptly communicates rationale for the decline of the referral to the PCP so that non-genomics-informed care can be initiated or continued. Communicating the rationale for the decline helps the PCP to build genomic reasoning over time and to improve the genomic assessments and referrals.
11. If the referral is considered appropriate for genomic care, then the triage team summarizes the phenotype and places the patient into a care stream based on urgency, best-fit CGS, and available subspecialty programs.
12. In preparation for an appointment, additional information is collected from the patient and family. Secure pre-appointment electronic patient modules can collect the following information: prenatal and perinatal history, growth and development history, medical history, current issues and concerns, family history, previously completed testing, and needs and goals. Such modules can educate the patient and the family on what to expect during their appointment, provide background information about genomics, collect consent forms, and collect photographs for medical use (i.e., to supplement patient assessment for remote appointments). Family-provided information is reviewed by the CGS in preparation for the appointment.
13. Guided by information from the PCP, the medical records, and the family, the CGS completes a clinical assessment.
14. The CGS generates a thorough and informative phenotype using HPO or similar terms that can guide genomic analysis and mitigate laboratory mining of clinical letters. The Genomic Testing Eligibility Assessment Tool (Supplementary Material 3) captures such phenotype and develops a justification for genomic testing.
15. The CGS determines whether genomic testing is indicated using biological evidence (Figure 1), and subsequently utility considerations (Figure 3B).
16. Should testing not be indicated, the CGS communicates the patient’s ineligibility for testing to the patient/family and the PCP. The CGS provides the PCP with instruction regarding clinical features to monitor for changes in eligibility.
17. Should testing be indicated, the CGS counsels and consents the patient and other family members receiving testing. The pretest counseling includes a) information about the test (the purpose of testing, the voluntary nature of genomic testing, the ability of the test to detect disease), b) potential limitations of testing, c) potential benefits and risks of testing (confidentiality protections and genomic discrimination risks and protections), d) potential genomic testing results (negative, positive, uncertain), e) unanticipated results (non-paternity, consanguinity, incidental finding), f) potential sample retention by the lab for internal research, g) privacy protections and access to sample and genomic information, h) cost of genomic testing and insurance preauthorization, and i) disclosure of results (phone vs. in-person, anticipated turn-around time).^1^
18. In requesting funding for testing, the CGS justifies the testing and delineates the eligibility criteria.
19. The payer reviews the funding request applying biological and utilitarian reasoning to decide whether to grant funding (Figures 1 & 3B). Should genomic testing be deemed unnecessary, the payer communicates the reasons for decline to the CGS. This can conclude CGS involvement (see 16).
20. If genomic testing is deemed appropriate, the payer communicates approval and makes funds available.
21. Once testing is approved, the CGS provides test requisitions to the patient and phenotypic information (e.g., HPO terms) to the laboratory.
22. The patient and any other family members being tested provide timely samples (typically blood or saliva) to ensure that the phenotypic data used in genomic analysis reflects the current patient phenotype.
23. The laboratory completes genome-wide sequencing (GWS), followed by alignment, variant calling, annotation, filtering, and variant classification (Figure 5).
24. Top candidate variants are further interpreted in the context of phenotype, medical history, and family history (Figures 4 and 5). To maximize accuracy and efficiency, variant interpretation is completed collaboratively by LGSs and CGSs. Collaboration can be synchronous (e.g., patient case conferences) or asynchronous (e.g., evidence building documents). Only the variants suspected to be disease-causing are included in the final clinical report.
25. Following interpretation of the reported variants, the CGS returns the testing results to the patient and their family members. Regardless of whether there is a finding, the patient receives post-test counselling. Patients without test findings are counselled that the outcome may be due to a lack of genomic cause or due to current limitations in technology and knowledge. Patients with genomic findings (i.e., a variant classified as variant of uncertain significance with high suspicion, likely pathogenic, or pathogenic) are informed of the nature and impact of the finding. Patients are provided with additional education and resources.
26. The CGS determines whether the patient health concerns are fully, partially, or not explained by the genomic findings.
27. Should a patient’s health concerns be fully or partially explained by a genomic finding, the CGS initiates genomics-informed care in collaboration with the PCP. This can include pursuing additional investigations (e.g., medical imaging) for surveillance of the body systems that are likely to be impacted by the disease, determining prognosis, and altering medications. The CGS provides guidance to the PCP in establishing and maintaining a care plan that caters to the genomic findings.
28. The PCP provides the patient with long-term care management and support. Additionally, the PCP monitors for changing or new symptoms that warrant a re-referral to the CGS. For patients with genomic findings, a change in phenotype might alter the interpretation of genomic variants. A marked phenotype evolution might qualify the patient for retesting.
29. If the patient’s concerns are not fully accounted for by the testing results, the CGS assesses the utility of further investigation (return to step 15).
30. If no further investigations are pursued, the CGS and the patient consider future reanalysis (Figures 1, 3A, 3B).
31. Automated variant-level re-evaluation is completed periodically. Whether to pursue variant-level and/or case-level reanalysis depends on phenotypic evolution. Re-sequencing is considered with advances in sequencing technologies.

# Supplementary Material Section 2. Prototyping an Electronic Platform for Genomic Medicine Using REDCap

Technology can be leveraged to increase interoperability, avoid redundancies, and increase patient throughput. In paper-based systems, collaboration is physically limited, and data redundancies overburden healthcare staff and providers and risk the safety and quality of patient care. To address these barriers, we prototyped and implemented an end-to-end electronic platform for genomic diagnostic assessment to render care more secure, accessible, effective, and efficient.

We repurposed an instance of research electronic data capture (REDCap) software^2^ for clinical use. REDcap “is a secure, web-based software platform designed to support data capture for research studies, providing 1) an intuitive interface for validated data capture, 2) audit trails for tracking data manipulation and export procedures, 3) automated export procedures for seamless data downloads to common statistical packages, and 4) procedures for data integration and interoperability with external sources.”( <https://www.project-redcap.org/> ) We designed a custom platform based on the BC Provincial Medical Genetics Program’s (PMGP) process models. We incorporated instruments that facilitate each stage of care and enable generation of curated data reports. We also engaged patients in their care through secure questionnaires and provided automated alerts. The functions of the custom platform were piloted within smaller clinical teams before being rolled-out to all staff at the PMGP. Healthcare provider and staff feedback was used to make iterative improvements to the platform.

In alignment with the Genomic Medicine Process Map, we developed the following instruments within the platform:

1. **Proband and Family Identifiers**

- Collects patient and family identifiers (date of birth, sex and gender, health record numbers, etc. ) and contact information.

1. **Referral and Triage**

- Records referral information.
- Facilitates electronic triage. Triage clinicians are provided with guidance in completing the form to ensure consistency. Logic is applied based on selections.
- Prints a triage summary sheet for the patient chart.

1. **Care and Communication Coordination**

- Collects information about the clinical team, including their contact information, which is to be included in automated emails and alerts.
- Captures whether the patient is involved in any clinical programs or pilot projects.
- Records information about the patient’s appointment.
- Facilitates triggering of automated patient alerts that may contain information about their appointment, links to pre-appointment questionnaires, educational modules, and reminders.

1. **Patient/Family Pre-Appointment Questionnaire**

***If your institution has REDCap, this instrument can be viewed and downloaded by searching for the “Genetics Pre-Appointment Questionnaire” instrument in the REDCap Shared Library:***

- Provides families basic information about genomics and the medical genomics service.
- Collects information about patient/family needs, health concerns, medical and family history, etc.
- Provides clinicians patient-completed information electronically or via a summary print-out.

1. **Photo and Consent Surveys**

- Allows patients to securely complete a medical photography consent form and to share photographs with their clinicians.

1. **Phenotype**

***If your institution has REDCap, this instrument can be viewed and downloaded by searching for the “Genetics Phenotype Assessment” instrument in the REDCap Shared Library***

- Captures phenotype in a way that is standardized and compatible with genomic analysis software (e.g., HPO terms).
- Records clinician assessment of patient phenotype, family history, and medical history.
- Populates certain fields based on the information the patient provided in the pre-appointment questionnaire.
- Creates pedigrees electronically.
- Generates a summary sheet. For the patient charts or for transmission to the testing laboratory.

1. **Genome Wide Sequencing Eligibility Form**

- Assesses patient eligibility for testing based on regional criteria.
- Generates a summary that can be sent to the funding agency.

1. **Genomic Testing Education Module**

- Summarizes important information and considerations for genomic and genomic testing in lay language.
- Collects families' concerns, questions, and preferences and sends a summary to the clinical team.

1. **Test and Outcomes Tracker**

- Documents testing details and status.
- Sends clinicians automated alerts to notify of funding expiry and delayed test reports.

1. **Clinical Variant Analysis Tool**

***If your institution has REDCap, this instrument can be viewed and downloaded by searching for the “Clinical Variant Analysis Tool (CVAT)” in the REDCap Shared Library***

- Archives genomic variants.
- Provides a framework for collecting and synthesizing evidence to ensure standardized clinical variant interpretation.

1. **Report generation**

The benefit to using an electronic information management platform is that information entered at the patient level can later be pulled to generate aggregate reports based on patient information and perform statistical analyses to inform clinic management:

- Referral and triage tracker
- Patient Questionnaire and Consent Completion Tracker
- Test Tracker
- Variant Tracker
- Specific trackers for clinical programs and cohorts.

Only certain instruments have been made publicly accessible through the REDCap Shared Library as the others are highly customized to the PMGP’s clinical processes. For those interested in learning more about the remainder of the instruments, please contact the corresponding author.

# Supplementary Material Section 3. Genomic Testing Eligibility Assessment Tool


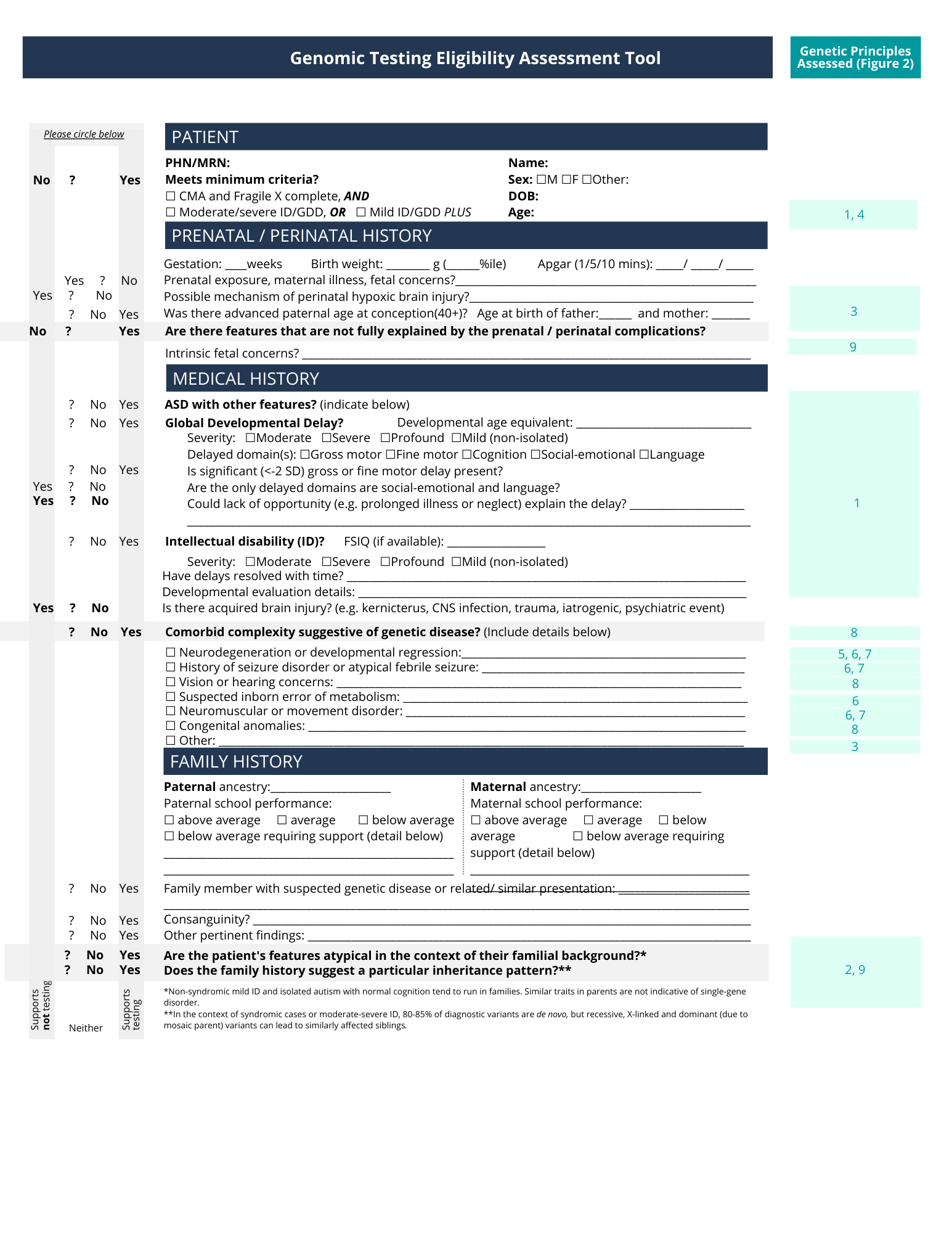


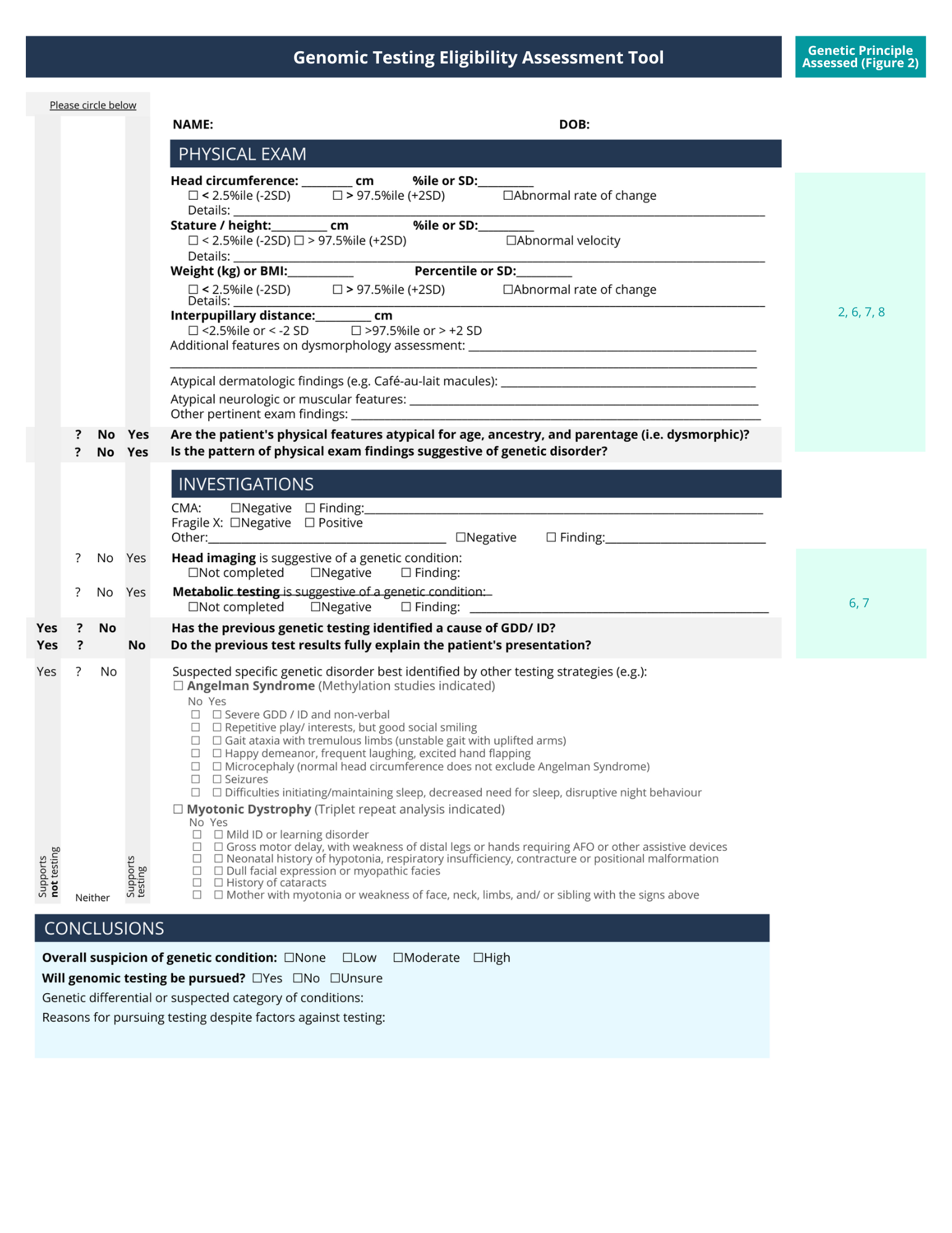


**INSTRUCTIONS FOR THE GENOMIC TESTING ELIGIBILITYASSESSMENT**

**BACKGROUND** This form serves as a tool with three objectives:

1. To evaluate the likelihood that the patient has a genetic disorder.
2. To evaluate the need for genomic testing (e.g., whole exome or genome sequencing).
3. To collect the pertinent information required for genomic analysis.

**HOW TO USE:** Circle one of "yes", "no","?" (unsure) left-hand column to answer the corresponding line item. Each option is placed based on whether or not the answer indicates that genomic testing should be pursued. Use the form sections under the dark headers to document relevant details.

**GENERAL STEPS** Establish that the patient meets minimum criteria: i) the patient is diagnosed with moderate-to-profound ID or GDD, or mild ID or GDD with additional features, and ii) both CMA and Fragile X testing are complete. If eligible, complete **all** form sections using information obtained from the medical records and during the appointment. **Provide an answer for all yes/no/? prompts.** Complete the "conclusion" section by assessing the placement of all circled answers.

**PRENATAL/PERINATAL HISTORY** assesses for any possible teratogenic exposures, concerns with fetal anatomy, movement, growth rate, or heart rhythm, maternal comorbid or acquired illness, mode of conception and mode of delivery. The timing of any concerns (obtain medical documentation if possible) should be noted. Prematurity and hypoxic injury are risk factors for brain injury. Birth weight should be plotted by gestational age. Advanced paternal age is associated with an increased rate of *de nova* variants in the offspring. To answer the bolded question, all information should first be integrated to generate an overall impression of the likelihood of acquired brain injury explaining the level of intellectual impairment.

**MEDICAL HISTORY**

- - Moderate to profound ID is more suggestive of a genetic disorder than is mild ID.
  - Multisystem involvement is more suggestive of a genetic disorder than is isolated ID.
  - Syndromic mild ID is more suggestive of a genetic disorder than is isolated mild ID.

Examples of pertinent details to note for the developmental assessment include the use of appropriate modifications for hearing or visual impairment, lack of patient motivation, and suspected underestimation of abilities

**FAMILY HISTORY:** For each **parent,** detail any learning difficulties, school supports, or modified curriculum. Inquire, as helpful, about parental occupation or level of education to assess the intellectual potential of the patient. For example, non­ syndromic mild intellectual impairment in the context of parents with above-average school performance could be suspicious for a genetic disorder if no other etiology for impairment can be shown. If the patient's measurements are outside of the typical range request heights or measure head circumference of parents. Examine mother for grip myotonia. It is appropriate to ask to look at photos of other family members to assess for familial facial features. To assess for **similarly affected siblings,** it is important to inquire about a family history of congenital anomalies, childhood illness/ death, and stillbirth. If such individuals exist, follow-up questions may inquire about previous genetic tests, autopsies, and diagnoses. Obtain medical records as indicated.

**PHYSICAL EXAM:** Measure head circumference, height, weight and interpupillary distance for all patients by standard methods. A standard pediatric exam should be performed. Neurological findings (including findings on fundoscopy) should be detailed in the indicated section. Dermatologic findings, which include hyper- or hypo-pigmentated macules, freckling, nodules, vascular lesions, and other skin qualities (wrinkling, extensibility, and fragility) should be detailed in the indicated section. Assessment may require Wood’s lamp (please indicate if used). Findings from cardiac, respiratory, and abdominal exams should be entered in "other pertinent exam findings."

**Dysmorphology Assessment** should note the presence of atypical:

| - Hairlines, head shape, hair whorl placement or number, hair, other quality, and face shape (front and profile) | - Body habitus, asymmetry to facial structure or part of body. |
| --- | --- |
| - Placement of ears (and posterior rotation}, ear shape, tags, pits, or creases. | - Length and width of the neck, neck pits, creases, cysts. |
| - Sparseness/ fullness of eyebrows, spacing of eyes, length of palpebral fissures, and quality of lashes. | - Chest shape, supernumerary nipples, pubertal changes, axillary pigmentation. |
| - Epicanthal folding and angle of palpebral fissures. | - Chest shape, supernumerary nipples, pubertal changes, axillary pigmentation. |
| - Length of nose, shape of nasal tip, and position of nares. | - Upper arm length, forearm length, carrying angle, supination/ pronation. |
| - Philtrum shape and length, mouth shape and size, cheek shape, and chin size and position. | - Hand/ foot size and shape, finger/ toe size and shape, thumb/ hallux position or shape, and nail size, thumb/ hallux position or shape, and nail size or quality. |
| - Coarse features and facial/ body hair distribution. | - Joint hypermobility or contracture, and scoliosis. |
| - Palate size and position, bifid uvula, oral frenulae, mucosal lesions, and tooth size, shape, and number. | - Genitalia and placement of anus |

For anything noted on dysmorphology assessment, comment if this is suspected to be familial and measure if possible.

# Supplementary Material Section 4A. Integration and evolution of biologic evidence across the process serves to determine the probability of a genomic diagnosis

The hypothesis is that the referred patient has a genomic disorder that explains the phenotype. A simplified depiction of four patient journeys is provided in the image below. The y-axis represents the suspicion (or probability) of a genomic disorder. The x-axis indicates time. Nodes represent sequential points in care across time: 1) referral, 2) triage, 3) appointment, 4) result disclosure, and 5) follow-up. Over time (x-axis), evidence (supporting and refuting the hypothesis) is collected. Horizontal dashed lines represent thresholds of suspicion required to advance to the next stage in care. In some clinical settings, such thresholds provide a rationale for referral and timing of genomic testing. For instance, a CGS may have sufficient data to offer genomic testing prior to seeing the patient. Note that “genomic diagnosis” refers to the general underlying suspicion for a single gene disorder or of a specific genomic diagnosis (illustrated with example A).

**
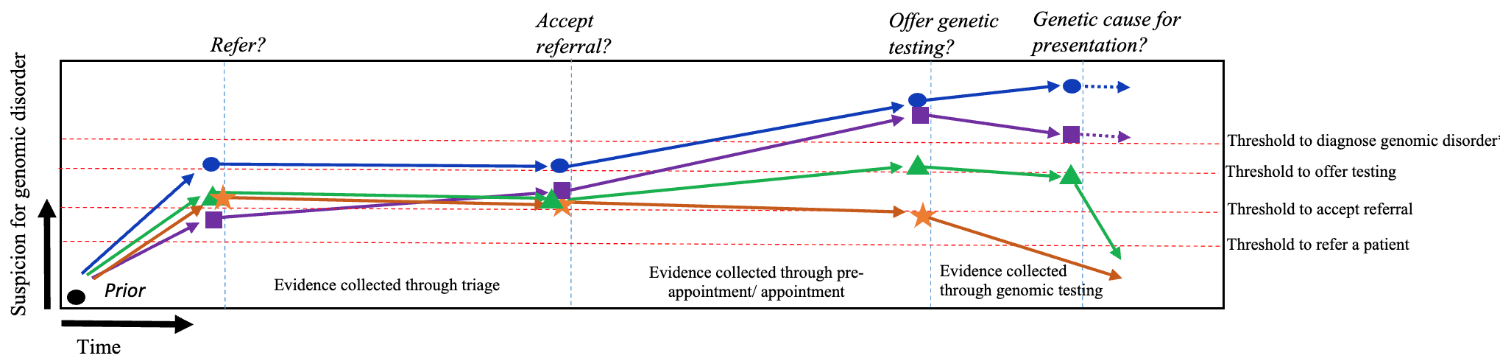
**

Patient A (blue circle): a 2 ½-year-old with moderate global developmental delay and a large ventricular septal defect that required repair. He is referred by his pediatrician for a possible single gene disorder. There were no in utero exposures or postnatal risk factors for poor neurodevelopment, and a chromosome array was normal (•1). The triage decision is to accept the patient as the information provided is sufficient (•2)_._ At his appointment, the history provided by the pediatrician is confirmed. There is no consanguinity, and there is no family history of similarly affected individuals. Physical examination reveals hypotonia, lower lid ectropion, long palpebral fissures, arched eyebrows, cupped ears, and persistent fetal fingertip pads consistent with Kabuki syndrome (•3). Genomic testing (trio whole exome sequencing) reveals a *de novo*, loss-of-function variant in *KMT2D* (•4)*.*

Patient B (purple square **■**): a 24-year-old with history of gestational diabetes that did not resolve after delivery, and evolved into insulin-dependent diabetes. Referred by the PCP for evaluation for MODY (pre-pregnancy BMI was 19) (**■**1). At triage, the family history revealed a strong history of diabetes in young adults; this followed an autosomal dominant inheritance pattern (**■**2). At appointment, the patient had a history of neonatal hypoglycemia, and at 5 years post-delivery, she still requires insulin to control her blood sugar. Recent work-up revealed the absence of beta cells antibodies and a C-peptide level in the normal range. Her physical examination was normal (**■**3). Broad panel testing reported no variants (**■**4). The CGS counsels the patient that MODY is still highly likely, and attributes the negative result to limitations in the current understanding of the contributory loci and the sensitivity of current technology. Enrollment in research is offered.

Patient C (green triangle ▲): a 2 ½-year-old with developmental delays and a unilateral cleft lip and palate. A recent hearing test was normal. She is referred by the pediatrician for evaluation for a possible single gene disorder after chromosomal microarray is normal (▲1). The triage decision is to accept the patient as the information provided is sufficient (▲2). At her appointment, it is confirmed that there were no in utero exposures. Developmental concerns are hyperactivity, delayed expressive speech (5 words), inconsistent response to name, temper tantrums, and poor fine motor skills. Gross motor development was advanced, and she enjoys chasing and copying her older siblings. There is no consanguinity, and there is a family history of ADHD, dyslexia, and dyscalculia. Examination reveals a single palmar crease on one hand, and no other pertinent findings ( ▲3). Genomic testing (singleton exome sequencing) reveals a VUS in *HDAC8*, a gene associated with X-linked Cornelia de Lange syndrome (▲4)*.* Segregation shows that the variant was inherited from a healthy mother; therefore, the VUS is felt to be non-contributory to the phenotype. The separate question of whether there is an underlying genomic cause is addressed by follow-up. Follow-up 18 months later reveals resolution of many developmental concerns with appropriate resources/ therapy. The updated clinical impression is that the cleft lip and palate are most likely isolated (non-syndromic) and of a multifactorial etiology. She is discharged from the genomics clinic with the appropriate family counselling (▲5).

Patient D (orange star **★**): a 1 ½-year-old with developmental delays and overgrowth. She is referred by the pediatrician for a possible single gene disorder after chromosome microarray is normal (**★**1). The triage decision is to accept the patient as the information provided is sufficient (**★**2). At her appointment, it is confirmed that she did not have gestational diabetes and that her prenatal ultrasounds did not detect fetal organomegaly. She was born at term weighing over 10 pounds, and postnatal growth has been at appropriate velocity. Developmental concerns are mild gross motor delay, no spoken words, and inconsistent response to name. There is no consanguinity, and there is a family history of increased birth weight and tall stature. Examination confirms that the height and weight are at +2.5 standard deviations (SD) and that the head circumference is at +2 SD. The CGS feels the overall clinical picture does not fit with an overgrowth syndrome and decides to follow up with the patient in 12 months without offering further testing (**★**3). At follow-up, the patient is developmentally appropriate with isolated tall stature, and discharged from the genomics clinic (**★**4).

# Supplementary Material Section 4B. Testing in the context of phenotype evolution.

The Y-axis (left) is representative of patient phenotype; a more severe and/or more complex phenotype is more indicative of an underlying genomic disorder (right Y-axis). The red horizontal dotted line indicates the threshold for suspicion of genomic disease at which genomic testing could be pursued. The X-axis is representative of time. Timepoint 1 refers to the present assessment of whether a patient should receive genomic testing. Timepoint 2 refers to a possible future assessment. During an appointment, the clinician must decide whether to pursue testing based on the historical and current phenotype. The area of the graph is greyed beyond Timepoint 1 to indicate that only medical history and present phenotype are available at the time of the initial assessment. Because the future evolution of the phenotype is unknown, genomic testing will never reach a diagnostic yield of 100% – there will always be cases in which the change in phenotype over time diminishes the probability of genomic disease (i.e., "false alarms").


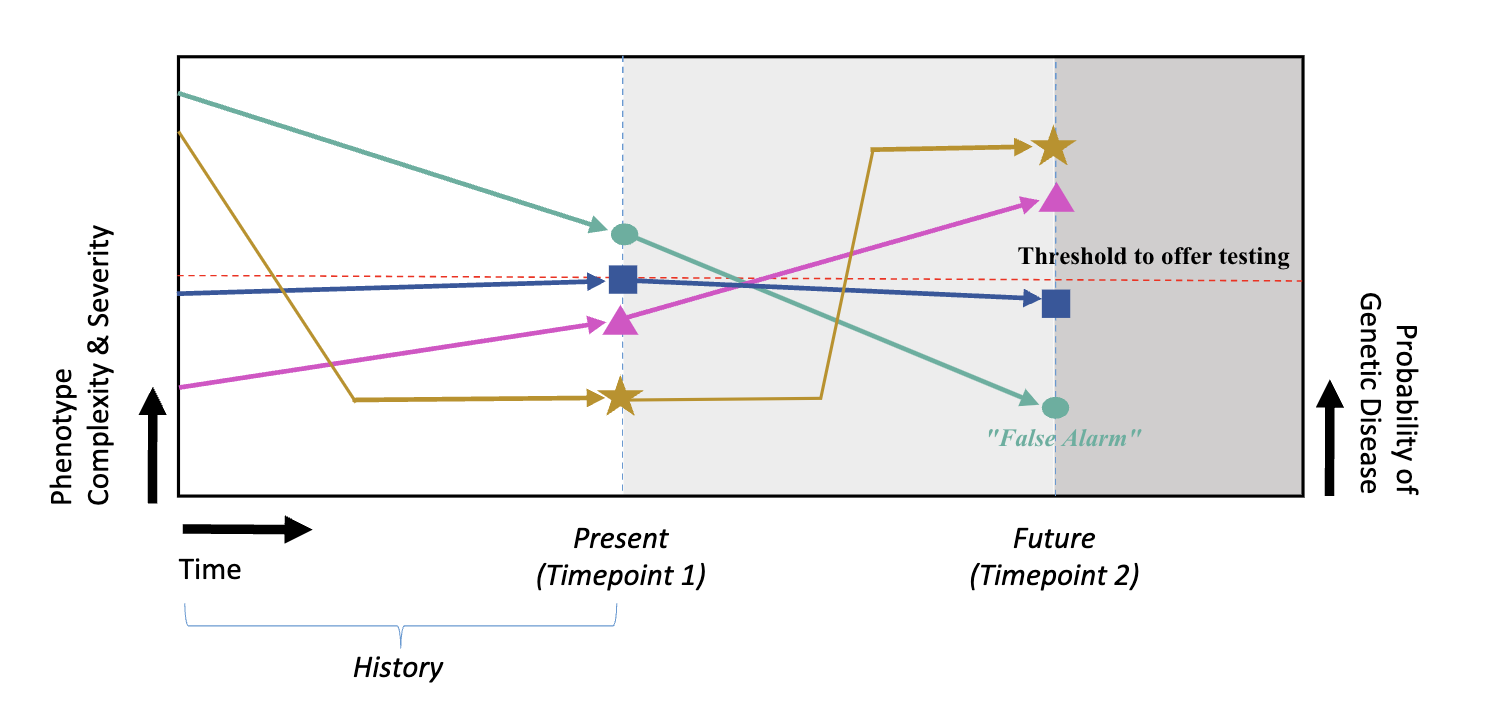


Case scenario A (pink triangle ▲): Phenotype severity and complexity are progressing over time: however, the suspicion of genomic disease does not meet the threshold for testing at Timepoint 1. The patient is monitored over time. At Timepoint 2, the phenotype has progressed beyond the threshold; therefore, the patient is offered testing. This might have been considered a "miss" or "false reject" if the patient had not been monitored, re-assessed, and provided with testing at Timepoint 2.

Case scenario B (green circle ●): Despite decreasing phenotype severity and complexity, the patient is provided with testing at Timepoint 1 because the suspicion of genomic disease and the utility of testing is above the threshold. Phenotype severity and complexity, however, decrease below the threshold and the suspicion of genomic disease is low at Timepoint 2. The patient's symptoms may resolve with no or little intervention. This would be considered a "False Alarm".

Case scenario C (blue square ■): Phenotype severity and complexity and suspicion of genomic disease are stable about the threshold for testing. The patient might be provided with genomic testing to clarify a differential or be monitored if the perceived benefit of testing is low.

Case scenario D (gold star ★): The patient had a history of disease which resolved without intervention and there is no need for testing at Timepoint 1. There is, however, episodic resurgence of disease severity/complexity. Although the symptoms resolve periodically, the suspicion of genomic disease remains high and warrants genomic testing.

# Supplementary Material Section 5. A grid representation of locus/disease-patient phenotypic concordance and specificity.


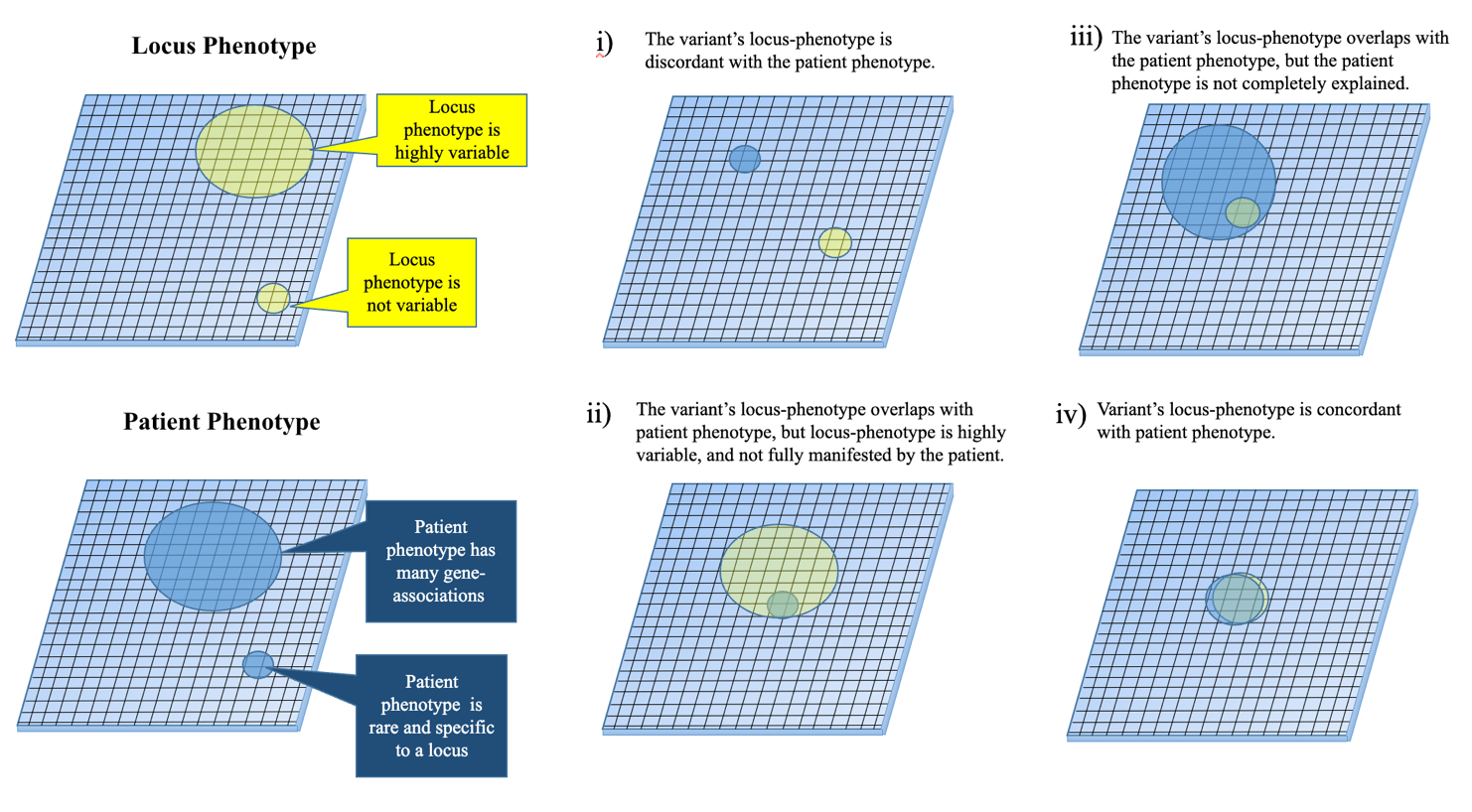


The blue grid represents possible phenotypes. In assessing the overlap between the phenotype associated with variation at a genomic locus (in yellow circles) and the patient phenotype (in blue circles), there are four general outcomes:

i) The variant locus phenotype is not variable, and the patient phenotype is specific; however, there is no or little overlap between the two. Specificity is high but concordance is low; therefore, the variant is unlikely to be disease-contributory.

ii) There is concordance between patient and variant locus phenotype; however, the variant locus phenotype is highly variable and not fully manifested in the patient. The variant may be disease contributory should variable expressivity be established.

iii) There is concordance between the patient and the variant locus phenotype; however, the patient phenotype is broader than the locus-associated phenotype. The variant may be disease contributory, but the patient phenotype is not fully explained by the variant.

iv) The variant locus and patient phenotype are highly concordant; therefore, the variant may be disease-contributory.

# Supplementary Material Section 6. Clinical Variant Interpretation Logic Applied

**CASE 1:** **Maternally-inherited missense variant in *ETV6***


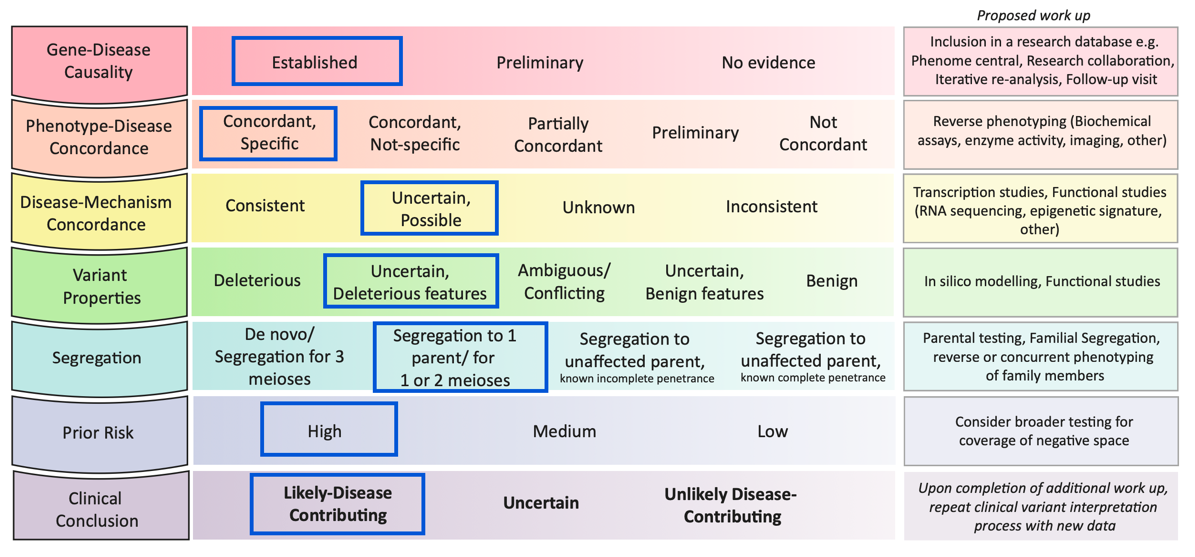


**Background & Phenotype (Prior Risk Assessment)*:*** A 10-year-old girl (proband) and her mother were seen by the CGS for childhood acute lymphoblastic leukemia (ALL). The maternal grandfather passed away from a myelodysplastic syndrome that progressed to acute myeloid leukemia (AML) at 55 years of age. The mother has ongoing thrombocytopenia and had genomic testing that ruled out a pathogenic *RUNX1* variant. The familial presentation was highly specific for *ETV6*-related disorder, a recently described disease locus that had not been included on the mother’s previous genomic testing. Whole exome sequencing with parental segregation reported a maternally inherited variant in *ETV6* missense variant in the proband. **Phenotype-Disease Concordance:** *ETV6* encodes a transcription factor regulating hematopoiesis. Heterozygous germline pathogenic variants in *ETV6* cause an autosomal dominant bone marrow disorder that manifests as thrombocytopenia and predisposes to ALL and other hematologic malignances. The familial phenotype is concordant with and specific for the disease phenotype. **Disease-Mechanism Concordance:** *ETV6* loss-of-function from frameshift and nonsense variants, exon deletions, and missense variants within the DNA binding domain predispose to disease. **Variant Properties:** The familial variant is absent in gnomAD and is located in the conserved ETS-domain that functions in DNA binding. The Grantham change was moderate (50-100). In silico tools predict the variant to be damaging with a CADD score of 30, and the crystal structure predicts the substitution to disrupt intra-molecular interactions. There is no crystal structure available to model the protein-DNA interaction and the effect of the substitution. Functional testing of DNA binding could support or refute the variant’s role in disease causation. The variant was classified as a variant of uncertain significance by ACMG criteria (PM2, PP3). **Segregation:** There were no other affected living individuals for further segregation studies. **Clinical Conclusion:** Clinically, the evidence was deemed sufficient to support a diagnosis and at-risk family members were offered molecular testing with concurrent CBC (given the high penetrance >90% of thrombocytopenia) for the familial variant.

**CASE 2: *De novo* loss-of-function variant in *NOTCH1***


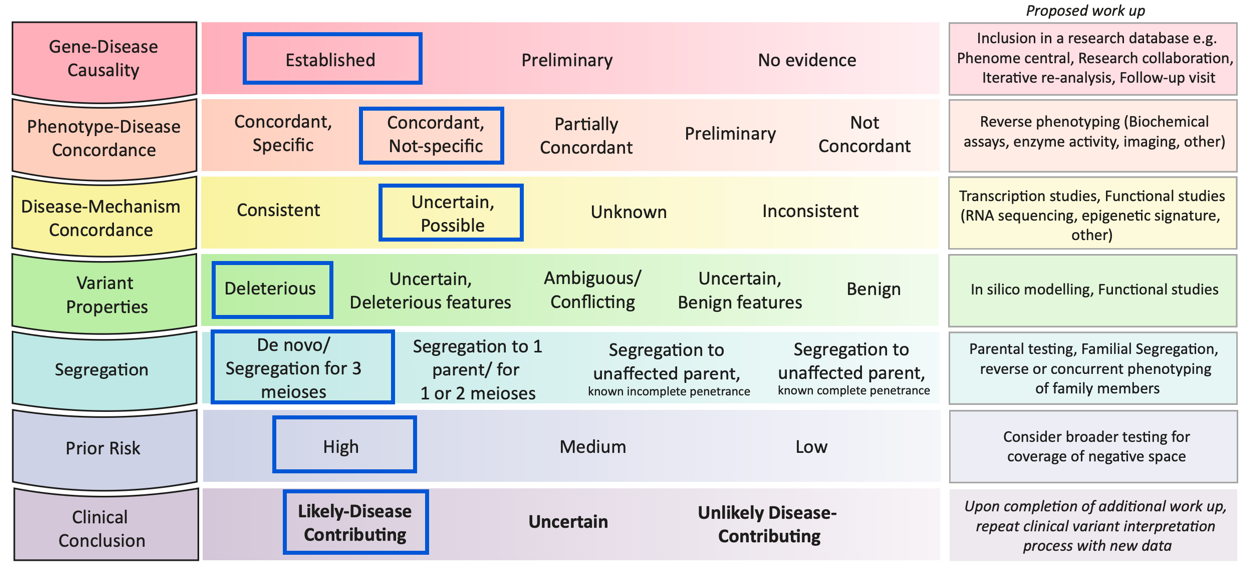


**Background & Phenotype (Prior Risk Assessment)*:*** The proband was born to parents without a personal or family history of cardiovascular conditions, recurrent miscarriages, or stillbirths. Aside from a 2-vessel umbilical cord, the first and second trimesters were unremarkable with noninvasive prenatal screening reported as low risk and a detailed anatomic ultrasound reported as normal. In the third trimester, a fetal ultrasound detected arrhythmia, heart block, and intrauterine growth restriction. The fetal arrhythmia persisted, and subsequent fetal ultrasound demonstrated severe symmetrical intrauterine growth restriction and cardiomegaly. Delivery was by emergent cesarean section. The proband died in the neonatal period from cardiorespiratory failure. Autopsy revealed multiple cardiovascular abnormalities. Trio whole exome sequencing demonstrated heterozygosity for a *de novo* likely pathogenic frameshift variant in *NOTCH1*. **Gene-Disease Causality:** In humans, haploinsufficiency of *NOTCH1* has been associated with cardiovascular malformation such as Adams-Oliver syndrome (AOS) and Aortic Valve Disease^3^. **Phenotype-Disease Concordance:** Deleterious *NOTCH1* variation is associated with congenital cardiac malformations, as seen in the patient^4^. Notch proteins are transmembrane receptors that play a key role in determination of cell fate. In mice, the absence of NOTCH1 results in malformation of the large blood vessels^5^. **Disease-Mechanism Concordance:** The variant in the proband results in the loss of function of NOTCH1, consistent with other reported disease-causing variants^6^. **Variant Properties:** The variant is absent in gnomAD, and predicted to result in a frameshift protein product incapable of transactivation. **Segregation:** The variant arose *de novo*. **Clinical conclusion:** Although the *NOTCH1* variant in the proband has not been previously reported, the clinical interpretation is that the variant is disease-contributing.

**CASE 3: Inherited 1.5 Mb copy gain at 17p13.3**


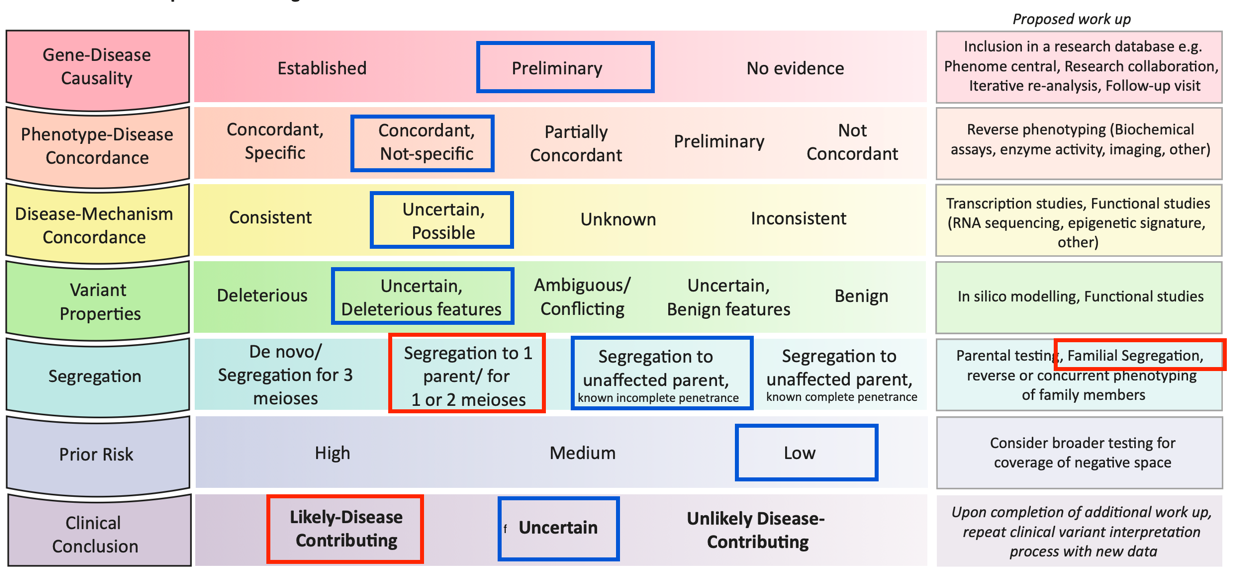


*Blue rectangles denote variant interpretation prior to segregation studies. Red rectangles reflect the changes to interpretation after familial segregation was assessed.*

**Background & Phenotype (Prior Risk Assessment)*:*** A 5-year-old boy with global developmental delay and autism had a chromosomal microarray, which reported a 1.5 Mb copy gain at 17p13.3 (not including *PAFAH1B1*) that the laboratory classified as pathogenic. **Gene-Disease Causality:** Similar copy gains of this region are reported to cause a variable effect on brain development (normal intellect, learning disorders and intellectual disability, and autism spectrum disorders).^8^ Penetrance is incomplete. The copy gains can arise *de novo or* be inherited from an unaffected parent. The locus-disease association was considered preliminary by the clinical team based on the few reported individuals and the absence of broad genomic testing to rule out other causes. **Phenotype-Disease Concordance:** The patient had a consistent but not a specific phenotype. **Disease-Mechanism Concordance:** The disease mechanism is unknown, but triplosensitivity of the brain-expressed *YWHAE* is a theoretical contributor. **Variant Properties:** Duplications that include *YWHAE* are absent in the Database of Genomic Variants. Smaller overlapping copy gains are present in the Genome Aggregation Database (gnomAD) at low frequency. **Segregation:** Segregation studies revealed that the copy gain was inherited from an unaffected parent and arose in the unaffected mosaic grandparent. **Clinical conclusion:** In the context of established incomplete penetrance and the recent *de novo* origin of the copy gain in the family, the variant is interpreted as disease contributing for the proband. Further testing was not arranged on the proband, as the copy gain explains the full clinical picture. The parents were counselled regarding the implications of the 17p13.3 copy gain and the limitations of molecular testing to predict incomplete penetrance.

# REFERENCES

1. Ormond K. *Defining the Critical Components of Informed Consent for Genetic Testing: A Delphi Study*.; 2021. doi:https://doi.org/10.1101/2021.06.24.21259406

2. Harris PA, Taylor R, Thielke R, Payne J, Gonzalez N, Conde JG. Research electronic data capture (REDCap)—A metadata-driven methodology and workflow process for providing translational research informatics support. *J Biomed Inform*. 2009;42(2):377-381. doi:10.1016/j.jbi.2008.08.010

3. Garg V, Muth AN, Ransom JF, et al. Mutations in NOTCH1 cause aortic valve disease. *Nature*. 2005;437(7056):270-274. doi:10.1038/nature03940

4. Dudoignon B, Huber C, Michot C, et al. Expanding the phenotype in Adams–Oliver syndrome correlating with the genotype. *Am J Med Genet Part A*. 2020;182(1):29-37. doi:10.1002/ajmg.a.61364

5. Krebs LT, Xue Y, Norton CR, et al. Notch signaling is essential for vascular morphogenesis in mice. *Genes Dev*. 2000;14(11):1343-1352. http://www.ncbi.nlm.nih.gov/pubmed/10837027.

6. Gerhardt DM, Pajcini K V., D’altri T, et al. The Notch1 transcriptional activation domain is required for development and reveals a novel role for Notch1 signaling in fetal hematopoietic stem cells. *Genes Dev*. 2014;28(6):576-593. doi:10.1101/gad.227496.113

7. McCarter AC, Wang Q, Chiang M. Notch in Leukemia. In: ; 2018:355-394. doi:10.1007/978-3-319-89512-3_18

8. Curry CJ, Rosenfeld JA, Grant E, et al. The duplication 17p13.3 phenotype: analysis of 21 families delineates developmental, behavioral and brain abnormalities, and rare variant phenotypes. *Am J Med Genet A*. 2013;161A(8):1833-1852. doi:10.1002/ajmg.a.35996
